# Unveil the Anticancer Potential of Limomene Based Therapeutic Deep Eutectic Solvents

**DOI:** 10.1038/s41598-019-51472-7

**Published:** 2019-10-17

**Authors:** Carolina V. Pereira, Joana M. Silva, Liliana Rodrigues, Rui L. Reis, Alexandre Paiva, Ana Rita C. Duarte, Ana Matias

**Affiliations:** 1 Instituto de Biologia Experimental e Tecnológica, Nutraceuticals and Bioactives Process Technology Lab., Oeiras, Portugal; 2 Instituto de Tecnologia Química e Biológica António Xavier, Universidade Nova de Lisboa, Oeiras, Portugal; 3 3B’s Research Group - Biomaterials, Biodegradable and Biomimetic, University of Minho, Headquarters of the European Institute of Excellence on Tissue Engineering and Regenerative Medicine, Avepark 4805-017, Barco, Guimarães Portugal; 4 ICVS/3B’s PT Government Associated Laboratory, Braga/Guimarães, Portugal; 5 LAQV/REQUIMTE, Departamento de Química, Faculdade de Ciências e Tecnologia, Universidade Nova de Lisboa, Caparica, Portugal; 6 The Discoveries Centre for Regenerative and Precision Medicine, Headquarters at University of Minho, Avepark 4805-017 Barco, Guimarães, Portugal

**Keywords:** Pharmaceutics, Green chemistry

## Abstract

Deep eutectic solvents have been recently reported as an interesting alternative to improve the therapeutic efficacy of conventional drugs, hence called therapeutic deep eutectic solvents (THEDES). The main objective of this work was to evaluate the potential of limonene (LIM) based THEDES as new possible systems for cancer treatment. LIM is known to have antitumor activity, however it is highly toxic and cell viability is often compromised, thus this compound is not selective towards cancer cells. Different THEDES based on LIM were developed to unravel the anticancer potential of such systems. THEDES were prepared by gently mixing saturated fatty acids menthol or ibuprofen (IBU) with LIM. Successful THEDES were obtained for Menthol:LIM (1:1), CA:LIM (1:1), IBU:LIM (1:4) and IBU:LIM(1:8). The results indicate that all the THEDES present antiproliferative properties, but IBU:LIM (1:4) was the only formulation able to inhibit HT29 proliferation without comprising cell viability. Therefore, IBU:LIM (1:4) was the formulation selected for further assessment of anticancer properties. The results suggest that the mechanism of action of LIM:IBU (1:4) is different from isolated IBU and LIM, which suggest the synergetic effect of DES. In this work, we unravel a methodology to tune the selectivity of LIM towards HT29 cell line without compromising cell viability of healthy cells. We demonstrate furthermore that coupling LIM with IBU leads also to an enhancement of the anti-inflammatory activity of IBU, which may be important in anti-cancer therapies.

## Introduction

Deep eutectic solvents (DES) have emerged in the last decade as a new class of ionic liquids (ILs) analogues^1–4^. Although DES share many characteristics with ILs, the terms are not interchangeable and DES offers several other advantages that turns them a viable alternative to ILs^1,4–6^. Contrary to ILs, DES fully obey the green chemistry metrics being less toxic, often biodegradable and no waste is generated upon their production^1,2,7,8^. Furthermore, DES are cheaper to produce, since the raw materials have lower cost and the synthesis is very simple and with high purity, when compared with other designer solvents^1,9^. DES are obtaining by mixing two or more components which at certain molar ratio supress the melting point of their chief compounds^1,6,10^. This depression in temperature is the result of charge delocalization occurring via hydrogen bonding between the components of the mixture^10–12^. The properties of DES can be tuned by changing the molar ratio and/or nature of hydrogen bond donor (HBD) and the hydrogen bond acceptor (HBA), which in turns influence the position and the number of the hydrogen bonds^3,13^. All these attractive features have positioned DES as attractive and advanced designer solvents with a wide range of applications including extraction, carbon dioxide capture, electrochemistry, biocatalysis and biomedical applications^9,10,14^. In the biomedical field it has been reported that DES improve the solubility, permeation and absorption of model active pharmaceutical ingredients (API’s)^10,15–18^. When one of the components of the DES is an API the system is hence called therapeutic deep eutectic solvents (THEDES)^16,17,19^.

Carcinogenesis is a phenomenon not only restrict to the abnormal growth of cells but also includes angiogenesis and inflammation processes, which play an important role in tumor progression^20^. The bioactivity of these emerging solvents is not yet well explored, nonetheless, literature has already reported cytotoxicity of ammonium- and choline chloride-based DES for several cancer cell lines^12,21^. Hence, the main purpose of this work was to ally anticancer and anti-inflammatory properties of natural occurring molecules such as terpenes and fatty acids and non-steroidal anti-inflammatory substances (NSAIDS) as potential chemotherapeutical DES.

Limonene is a cyclic monoterpene studied at preclinical and clinical levels due to its chemopreventive and chemotherapeutic activities at several types of cancer, as lung, breast, gastric, prostate, etc^22,23^. It is recognized as safe (GRAS) being used at food industry as a flavoring agent^24^. Its high lipophilicity contributes to a favorable cellular absorption, specifically at intestinal level, leading to a good bioavailability in the systemic circulation^25^. In colorectal cancer, limonene has been reported to induce apoptosis via mitochondrial pathway and affect PI3K/Akt signaling pathway (survival and apoptosis)^26^. Regarding inflammation process, fatty acids, terpenes and NSAIDs have already been reported, where (i) saturated fatty acids (myristic acid (MA) and capric acid (CA)) are known to act against oxidative stress and pro-inflammatory cytokines, (ii) menthol is capable of reducing IL-1ß at chronic colonic inflammation, and (iii) ibuprofen (IBU) is a commercialize anti-inflammatory compound also associated tumor reduction^27–30^. The major goal of this work is to provide clues for future development of new pharmaceutical systems based on DES which may enhance the bioactivity and efficacy of the designed systems and can lead to significant breakthroughs in cancer therapy.

## Results

### Design and characterization of THEDES

In this work, LIM was mixed with different compounds (i.e., MA, CA, menthol IBU) to obtain novel formulations with pharmaceutical applications that may boost the use of THEDES in cancer treatments. The different molar ratios and visual aspect at room temperature (RT) are listed in Table 1. All the formulations based on MA (C14) yielded a pasty like solid and were readily discarded. Using CA:LIM at a molar ratio of 1:1; 1:2 and 2:1 a clear and transparent liquid without any precipitate was obtained at RT after 1–2 hours. Similarly using menthol:LIM no insoluble particles were visible at naked eye and, thereby, a clear liquid was obtained. Thereby, the equimolar ratios of menthol and CA based THEDES were selected to evaluate the cytotoxicity. LIM was also combined with IBU at different molar ratios, either equimolar or imbalanced. The molar ratios of 1:4 and 1:8 were the only ones where a liquid was obtained at RT. However, it should be noted that with a molar ratio of 1:4 small crystals were observed at RT.

**Table 1:** Summary of the different THEDES prepared.

THEDES	Molar Ratio	Visual aspect at RT	Melting Point (°C)
MA:LIM	1:1 1:2 2:1	Solid Solid Solid	≈47.7 ≈47.5 ≈51.6
CAl:LIM	1:1 1:2 2:1	Liquid Liquid Liquid	≈14.3 ≈7.8 ≈20.9
Menthol:LIM	1:1 1:2 2:1	Liquid Liquid Liquid	— — —
IBU:LIM	1:1 1:2 2:1 4:1 8:1	Solid Solid Solid Liquid with few crystals Liquid	≈58.4 ≈59.8 ≈63.05 ≈29.8 —

**Figure 1:**
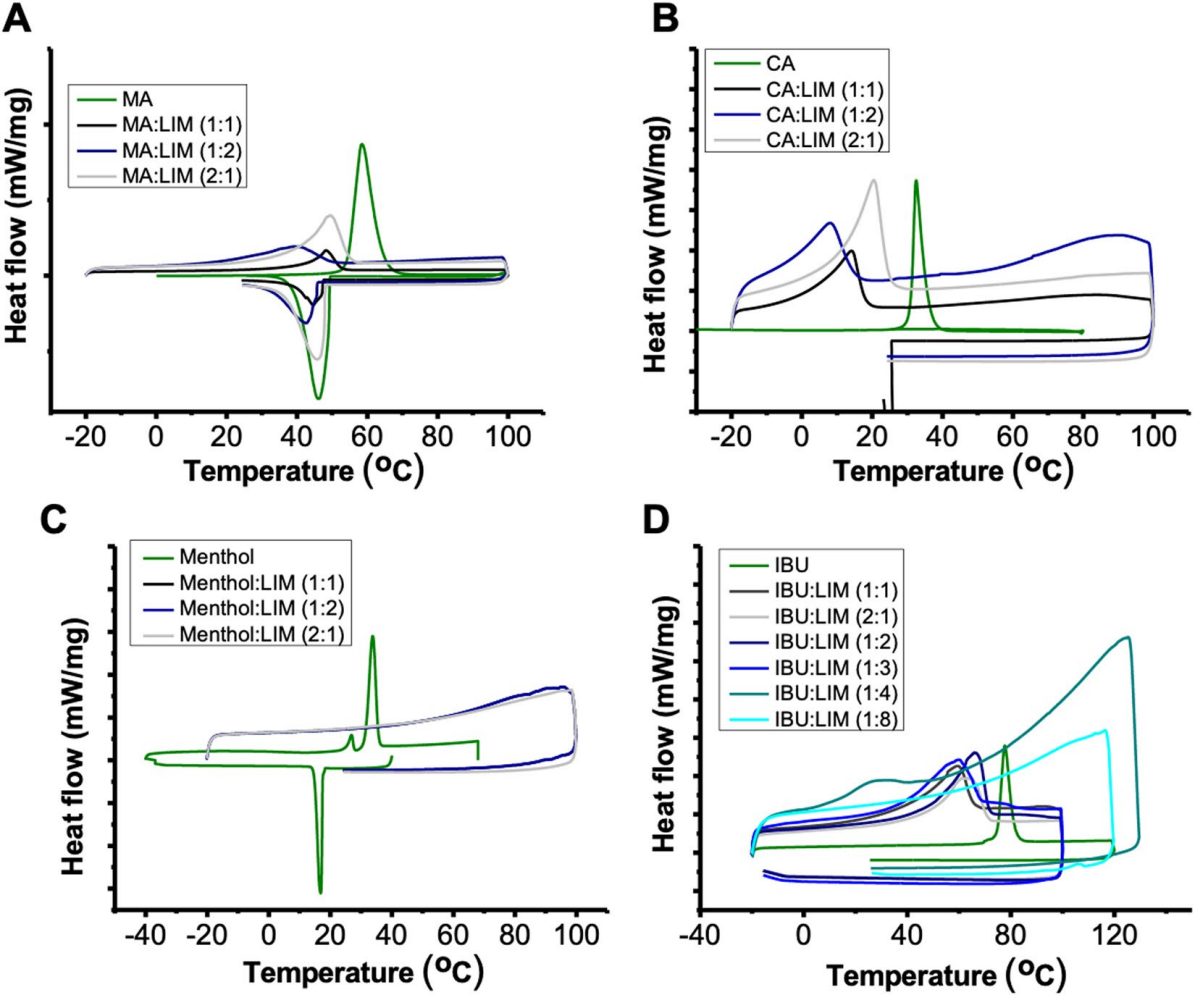
DSC thermograms of powders and THEDES based on LIM: (**A**) MA:LIM, (**B**) CA:LIM, (**C**) Menthol:LIM, (**D**) IBU:LIM. Peaks arising above the baseline represent endothermic peaks.

THEDES were further studied by differential scanning calorimetry (DSC) to assess thermal events. The MA:LIM spectra present a unique and well-defined peak at the different molar ratios, which range from ≈ 51.6 °C to ≈ 47.5 °C. In all the molar ratios a depression on the melting point of MA≈ 58.6 °C was observed (Fig. 1A). Similarly, using CA (C10) a unique and well-defined peak was observed at all the molar ratios (Fig. 1B), which is far lower than the ones of THEDES based on MA (C14) as well as from the CA powder ≈ 31.5 °C. On the other hand, the spectra of menthol:LIM at different molar ratio does not present any peak from the alpha and beta polymorphs of menthol (≈ 28 °C and ≈ 33 °C), (Fig. 1C). Likewise, LIM was also mixed with IBU at different molar ratio and the thermograms were different depending on the molar ratios. The thermograms at molar ratios  from 1:1 until 1:3 were dominated by the presence of sharp endothermic peaks, that are ascribed to IBU. Contrarily, using a molar ratio of 1:4 and 1:8 a strong depression on the melting point of IBU was observed (Fig. 1D).

### Assessment of cytotoxicity and antiproliferative effects of LIM-based THEDES

**Figure 2:**
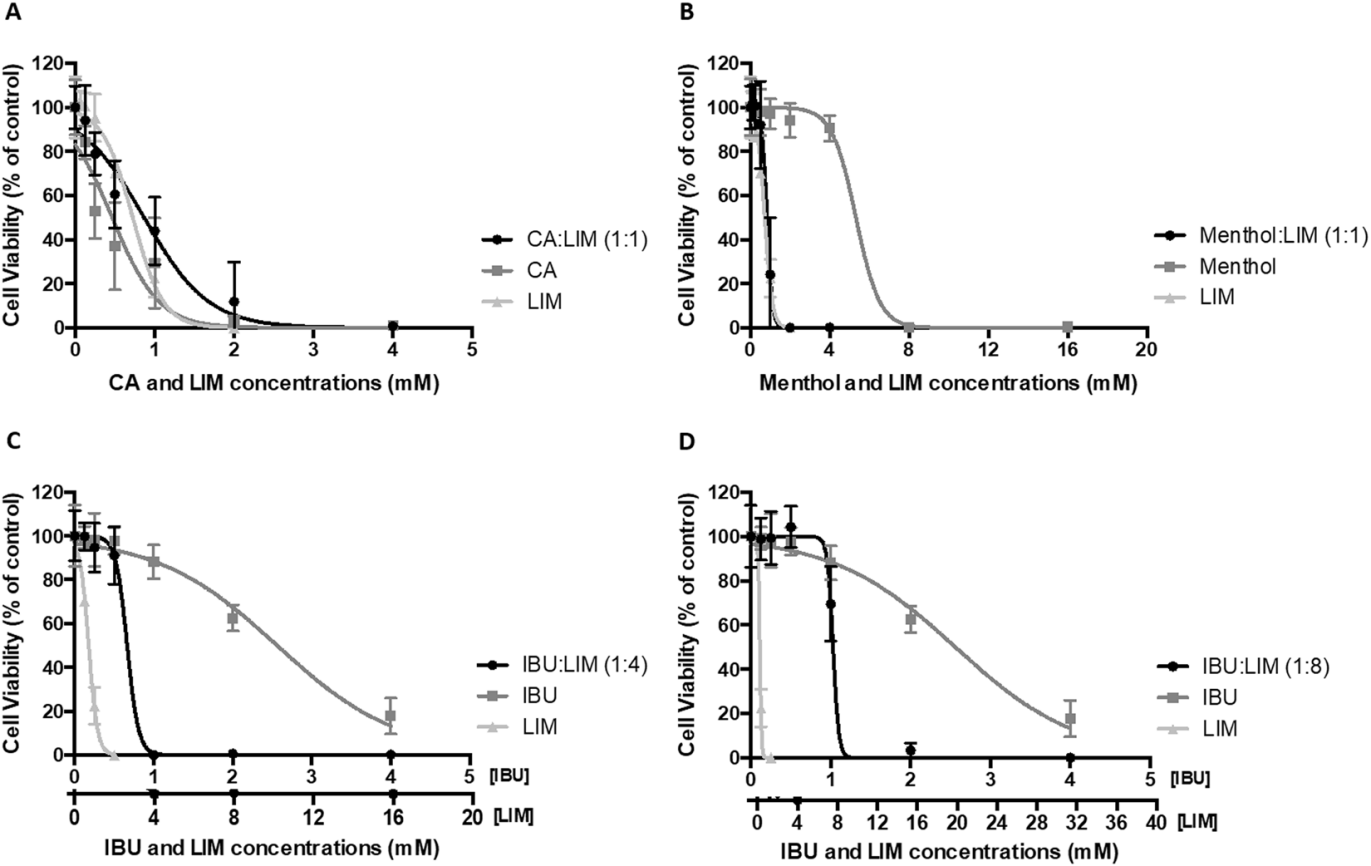
Antiproliferative effect of (**A**) CA:LIM (1:1), (**B**) menthol:LIM (1:1), (**C**) IBU:LIM (1:4) and (**D**) IBU:LIM (1:8) using HT29 cell model. Results were expressed relatively to the control as mean ± SD of at least three independent experiments performed in triplicate.

LIM-based THEDES were evaluated in terms of their cytotoxicity and antiproliferative effects. The cytotoxicity was assessed with Caco-2 cell line in a confluent and non-differentiated cell monolayer after 7 days of culture. After this time, this cell model shares some characteristics with crypt enterocytes, and thus it has been considered as an accepted intestinal model widely implemented to assess the toxicity of chemicals, food compounds and nano/microparticles on the intestinal epithelial cells (IECs)^31–33^. On the other hand, HT29 cell line was used to evaluate cancer cell proliferation, where after 24 hours seeding, the treatment with DES was performed in cancer cells at their growth/proliferative phase. As Fig. 2 shows, CA:LIM (1:1), menthol:LIM (1:1), IBU:LIM (1:4) and IBU:LIM (1:8) systems inhibited HT29 proliferation in a dose-dependent manner, being CA:LIM (1:1) system the one with lowest EC_50_ value (0.6901 ± 0.105 mM of equivalent LIM, Table 2).

**Table 2:** EC_50_ values obtained from cytotoxicity and antiproliferative assays of isolated compounds and THEDES in Caco-2 and HT29 cells, respectively. Results were expressed in mean ± SD of equivalents of LIM.

Isolated compounds	EC_50_ values (mM)	
	Cytotoxicity assay	Antiproliferative assay
IBU	2.893 ± 0.059	2.346 ± 0.088
CA	1.334 ± 0.223	0.341 ± 0.081
LIM	2.638 ± 0.108	0.661 ± 0.025
Menthol	8.078 ± 0.810	4.730 ± 16.14
THEDES	***equivalents of Limonene (mM)***	
CA:LIM (1:1)	0.918 ± 0.042	0.6901 ± 0.105
Menthol:LIM (1:1)	2.314 ± 0.421	0.8023 ± 0.016
IBU:LIM (1:4)	10.50 ± 0.883	2.390 ± 2.919
IBU:LIM (1:8)	3.323 ± 0.228	1.137 ± 0.055

CA:LIM (1:1) and menthol:LIM (1:1) systems showed similar antiproliferative activity comparing to isolated LIM (Table 2). Using IBU:LIM (1:4) and IBU:LIM (1:8) eutectic systems a distinct behaviour was observed. Although, all THEDES showed antiproliferative effect, only IBU:LIM (1:4) was selected for further bioactivity evaluation. To further explore the distinctive properties of the THEDES prepared it is crucial the comparison of IBU:LIM (1:4) effect with the simple physical mixture of IBU and LIM. In this case, the objective is to evaluate if the a priori preparation of the THEDES, in which there is a supramolecular arrangement between IBU  and LIM and the two components which are not interacting and were dissolved in the liquid media at the same concentrations influences the results.

**Figure 3:**
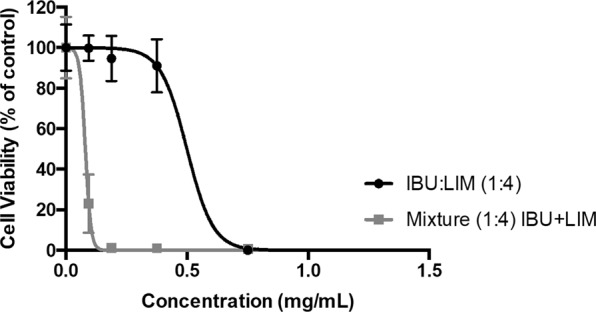
Comparing antiproliferative effect of THEDES and a mixture of IBU and LIM. IBU:LIM (1:4), and a mixture of IBU and LIM were compared in terms of antiproliferative activity using HT29 cell model treated for 24 hours. Results were expressed relatively to the control as mean ± SD of three independent experiments performed in triplicate.

As Fig. 3 shows, the physical mixture has higher antiproliferative effect (EC_50_ = 0.074 ± 0.006 mg/mL) and a clearly different behaviour from IBU:LIM (1:4) THEDES (EC_50_ = 0.4489 ± 0.548 mg/mL).

### Assessment of IBU solubility in LIM based THEDES

**Figure 4:**
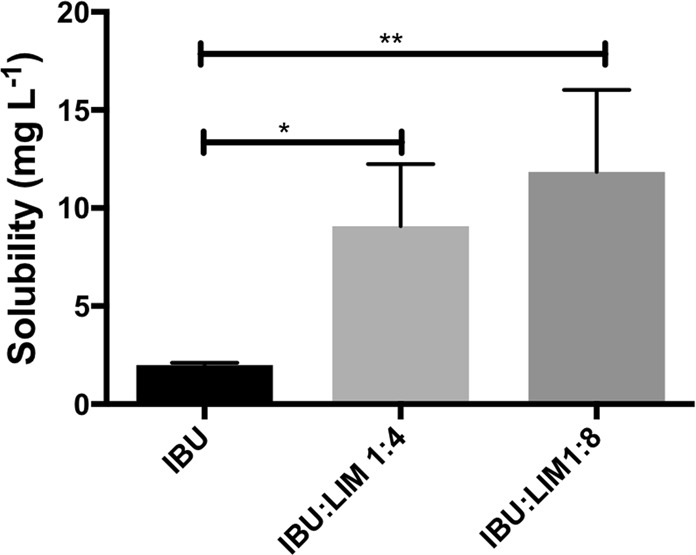
Solubility of IBU in powder form or complexed in THEDES in HBSS solution at physiologically like conditions (pH 7.4., 37 °C).

The potential of LIM based THEDES to increase the solubility of IBU was explored, as the therapeutic effectiveness of any API is strongly dependent of its solubility. As a non-steroidal anti-inflammatory IBU presents a very low solubility in water (≈ 21 mg L^-1^)^16,34^. Thus, the solubility of IBU in physiologically-like condition (i.e., HBSS at 37 °C) in powder and THEDES form was quantitatively determined (Fig. 4). The results clearly indicate an increase in IBU solubility by 4.32-fold (IBU:LIM (1:4)) and 5.63-fold (IBU:LIM (1:8)), when compared with IBU in powder form. The increase on its solubility while in THEDES form prevents its precipitation and the formation of visible aggregates on the bottom of vials.

### Bioactivity of THEDES

**Figure 5:**
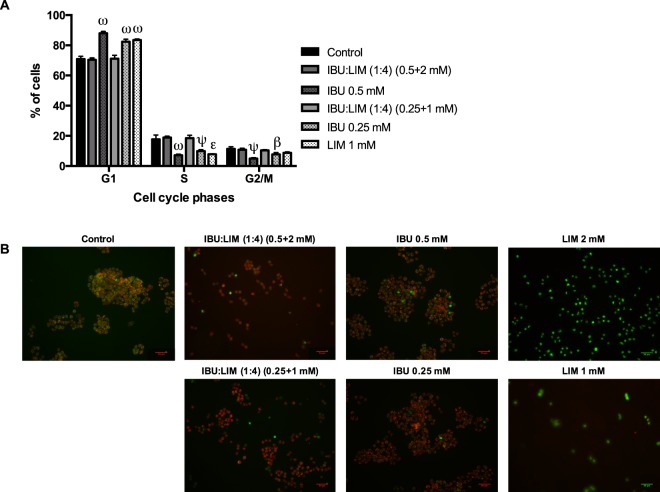
Effect of IBU:LIM (1:4), IBU and LIM in inducing cell cycle arrest and apoptosis on HT29 cells. Cell cycle analysis distribution on HT29 cells after incubation for 24 hours (**A**). Caspase-3 detection on HT29 cells treated during 24 hours. Active caspase-3 was detected by incubation with NucView488TM (apoptotic cells in green) and viable cells detected by incubation with MitoView633TM (cells with active mitochondria in red) (**B**). The scale bar is 50 μm. Results are mean ± SD of four (**A**) and two (**B**) independent experiments performed in duplicated. Statistical significant differences are expressed in asterisks (β = P ≤ 0.05, ε = P ≤ 0.01, ψ = P ≤ 0.001, ω = P ≤ 0.0001) by one-way ANOVA for multiple comparisons by Dunnett’s method.

Two non-cytotoxic concentrations of IBU:LIM (1:4) were used for analysis of cell cycle, apoptosis, intracellular ROS and NO production. As Fig. 5 shows, IBU:LIM (1:4) was not capable of inhibiting cell cycle nor apoptosis, whereas isolated IBU and LIM promoted cell cycle arrest in G1-phase (Fig. 5A). Additionally, LIM at 2 mM was capable of inducing apoptosis via caspase-3 activity (Fig. 5B). It should be noted that LIM at 2 mM was not tested for cell cycle assessment for being a cytotoxic concentration, as aforementioned.

**Figure 6:**
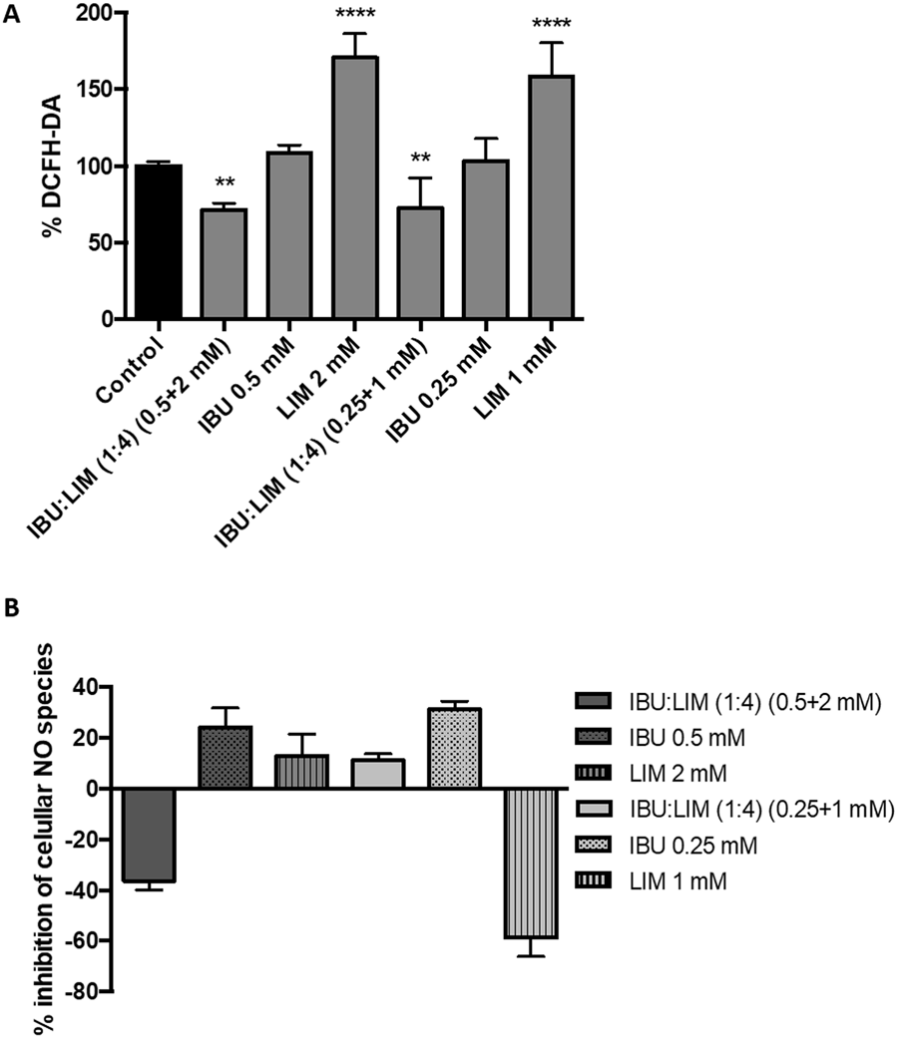
Effect of IBU:LIM (1:4), IBU and LIM on ROS accumulation (**A**) and NO production (**B**) using HT29 cell model with 24 hours treatment. Results were expressed relatively to the control as mean ± SD of three independent experiments performed in triplicate. Statistical significant differences were calculated according to one-way ANOVA for multiple comparisons by Dunnett’s method (** = P ≤ 0.01, **** = P ≤ 0.0001).

The data of oxide species production indicates that IBU:LIM (1:4) was capable of protect HT29 cells from oxidative stress by decreasing the production of basal intracellular ROS relatively to the control (Fig. 6A). Figure 6B shows that the highest concentration of IBU:LIM (0.25 + 2 mM) induced the production of NO, whereas the lowest concentration of THEDES (0.25 + 1 mM) protected HT29 cells from oxidative stress.

**Figure 7:**
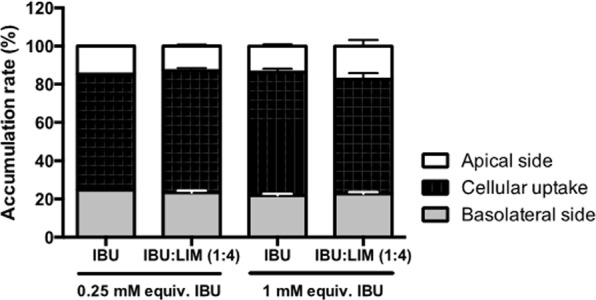
IBU:LIM (1:4) cell transport using differentiated Caco-2 transwell cell model. Results were expressed in terms of percentage of accumulation rate at the apical and basolateral sides of the transwell plate and cellular uptake. Statistical significant differences comparing to the same concentration of isolated IBU were calculated according to one-way ANOVA for multiple comparisons by Dunnett’s method.

Additionally, transport studies were performed using confluent and differentiated Caco-2 cell model. Figure 7 shows that IBU:LIM (1:4) had similar transport behaviour as IBU (no statistical differences) being mostly uptake by Caco-2 cells rather than transport from the apical side to the basolateral side of the transwell plate.

## Discussion

Herein, the main goal was the development of novel THEDES based on LIM due to its well-known and remarkable anticancer properties^35^. LIM was combined with different compounds, including saturated fatty acids (i.e., MA and CA), essential oil derivatives (i.e., menthol), and nonsteroidal anti-inflammatory drug (NSAID) (i.e., IBU) due to their well-known anti-inflammatory properties^29,36^ and/or improvement on the bioavailability of the API^37^. To achieve such goal, different formulations based on LIM were prepared using different molar ratios, either equimolar or imbalanced. The formulation that led to a pasty-like solid at RT were readily discarded, such as the ones based on MA (C14). These data suggest that the size of saturated chain strongly influenced the supramolecular arrangements, and, consequently, the depression on the melting points of starting pure materials (Fig. 2A). The depression on the melting point is highly dependent on the nature of the counterparts, molar ratio, lattice energy of DES and also entropy changes arising from the supramolecular arrangements^1,38–40^. DSC analysis confirmed the results of simple naked-eye observation, as a small decrease on the melting point of MA was observed. Using a saturated fatty acid with lower chain size, such as CA (C10), in equimolar and imbalanced ratios after half an hour a clear and transparent liquid was obtained at RT which was reflect by a depression on the melting point of CA. Using menthol as counterpart of LIM a transparent liquid was obtained at all the molar ratios, and the thermograms reveal the inexistence of any sharp peak of the menthol polymorphs (i.e., ≈ 28 °C and ≈ 33 °C). These peaks have been ascribed to alpha and beta polymorphs, being in good agreement with the literature^41–43^. Upon combining IBU with LIM at equimolar and imbalanced molar ratios up to 1:3, a slight depression on the melting point of IBU was observed, which suggested that depending on the molar ratios the shift is clearly different as one component will be in excess or default to stablish the intermolecular interactions. Using a molar ratio of 1:4 a strong depression on the melting point was observed and doubling the molar ratio the peak ascribed was completely suppress. Thereby, these molar ratios were the only ones that succeed in the formation of a liquid at RT and or near physiological temperature. It should also be noted that the thermograms of each individual starting materials corroborated previous data in the literature^41–45^. Additionally, the peaks obtained in DES are different from the ones of the parent species and a clear depression in the melting point occurs which further suggests the supramolecular rearrangement and the loss of lattice arrangement while the compounds are in DES form.

Combinations based on CA:LIM (1:1), menthol:LIM (1:1), IBU:LIM (1:4) and IBU:LIM (1:8) were the selected combinations of THEDES that were used to assess cell cytotoxicity and antiproliferative effects. The similar behaviour between LIM and CA:LIM (1:1) and menthol:LIM (1:1) (Fig. 2A,B, Table 2) suggests that the effect of these THEDES is mainly due to the action of LIM itself. LIM has been reported in the literature as a compound with antiproliferative properties by inhibiting colorectal cancer cell growth^26,46^. On the other hand, IBU:LIM (1:4) and IBU:LIM (1:8) systems (Fig. 2C,D) have IBU which might have led to decrease antiproliferative activity of the systems comparing to LIM. Although, the results indicate that all the THEDES presented antiproliferative properties, IBU:LIM (1:4) was the only system able to inhibit HT29 proliferation without comprising cell viability (i.e., with the lowest cytotoxicity for normal colonic cells (Caco-2 model)), as shown in Table 2 Therefore, IBU:LIM (1:4) was the formulation selected for further assessment of anticancer properties.

The hypothesis of having similar or different effects between THEDES and a simple physical mixture of the compounds has also been raised in this work. A direct mixture of IBU with LIM in culture medium was performed and antiproliferative activity assessed showed to be totally different from IBU:LIM (1:4) (Fig. 3), confirming once more the supramolecular arrangement whereas in THEDES form. These screening tests allow the preparation of systems where it will be possible to take advantage of the interactions effect between the individual components. Additionally, using LIM combined with IBU an increase of solubility of 4.32-fold occurs when compared with IBU in powder form. An increase on the solubility of IBU prevents its precipitation and coagulation, which is a step forward to accomplish desire pharmacological responses^47,48^. Similar results were obtained using menthol combined with IBU^16,18^. The strong solubilization of poorly water-soluble compounds while in THEDES form is referred in the literature as a phenomenon called hydrotropy. Hydrotropes are described as molecules able to enhance the solubility of hydrophobic molecules in water by means other than micellar solubilization^49–51^. This is in fact a major advantage of the DES which can be further explored not only for pharmaceutical but for other fields of application^15–18,52–56^.

Anticancer properties of IBU:LIM (1:4) were afterwards evaluated analysing the effects on cell cycle, apoptosis, intracellular ROS and NO production. The fact of IBU:LIM (1:4) was not capable of inhibiting cell cycle nor apoptosis suggests the hypothesis of the antiproliferative effect of IBU:LIM (1:4) followed another mechanism of action than the cell death by caspase-3 dependent apoptosis or cell cycle arrest. Studies in the literature reported anticancer properties by modulation of signalling molecules such as reactive oxidative species (ROS), nitric oxide (NO) and NF-kB pathway^57^. Assessing intracellular ROS and NO production, the results indicate that the lowest tested concentration of IBU:LIM (1:4) (0.25 + 1 mM) protected HT29 cells from oxidative stress, demonstrating anti-inflammatory effects by inhibiting ROS and NO production (Fig. 5A,B). Inflammation is a process intrinsically associated with carcinogenesis and, consequently, NO levels were determined since they play an important role in modulating the inflammatory molecular pathways, being highly increased in human colonic mucosa^58^. Moreover, an inappropriate NO production has been also related with programmed cell death by oxidative stress^59^. Herein, the results showed a concentration dependence of IBU:LIM (1:4), where the highest concentration (0.5 + 2 mM) of THEDES promoted NO production above the basal intracellular level (although decreasing intracellular ROS level), leading to cell death. The apoptosis was not induced by caspase-3 cascade as shown in Fig. 5B, which suggested that this process should be induced via caspase-2 or caspase-9 pathways^59^. As a NSAID, IBU present anti-inflammatory and antiproliferative properties, which can lead to cell cycle arrest and induction of apoptosis^60–62^. The present results corroborated the ones in the literature and differences might be explained by different concentrations herein tested as well as to the cell lines.

IBU has been well-reported in literature as a compound easily absorbed at the intestinal gut by passive transport associated to pH gradient^63^. Terpenes, such as LIM, are known to stimulate paracellular transport of large molecules by interacting with tight junctions, however, its own transport is mainly passive^64^. For these reasons, transportation studies were performed using Caco-2 cell model in a transwell plate. The maintenance of TEER values (data not shown) reinforces the non-cytotoxicity of the tested concentrations. The results obtained indicate a transcellular transport of IBU:LIM (1:4), and their accumulation and metabolization inside the cells, which confirm the effectiveness IBU:LIM (1:4) action.

In summary, these results suggest that IBU:LIM (1:4) has different effects depending on the dose, being the mechanism of action completely different from isolated IBU and LIM. Therefore, these evidences demonstrate the effectiveness of the IBU:LIM (1:4) when compared with counterparts. Several studies in the literature reported that the hydrogen-bonded supramolecular arrangements stablished in DES may led to synergetic or additive effects between the counterparts^12,21,52,65–67^. In the beginning, the cytotoxicity of DES was mostly associated to the toxicity data of the counterparts. However, nowadays several studies highlight the possibility of synergetic or additive effects on the counterparts, which may have a strong impact on the biological performance^7,12,21,65–69^. It should be noted that the synergetic/additivity effects results in some cases in more or less toxic systems in comparison with their constituents^70^. Up to the date few data has been reported dealing with the cytotoxicity of DES, serving the obtained data to predicted the pharmaceutical potential applications of such systems, as it would not only be dependent on the nature of the chemicals used but also on the DESs formed. Indeed, this THEDES comprises the protective and anti-inflammatory properties of IBU allied to the anticancer properties of LIM. Thus, using IBU:LIM (1:4) it is possible to decrease the cell cytotoxicity associated with LIM and increase the solubility of IBU, which further show the potential of this system as a drug delivery system in anti-cancer therapies.

## Methods

### THEDES preparation

The reagents used in the preparation of THEDES were S-Limonene (LIM, ref.218367 Sigma Aldrich), myristic acid (MA; ref.70082, Sigma Aldrich), Capric acid (CA, ref. A14788.30, Sigma Aldrich), menthol (ref.M2772, Sigma Aldrich) and Ibuprofen (IBU, ref. I4883, Sigma Aldrich). THEDES of MA:LIM, CA:LIM, menthol:LIM and IBU:LIM were prepared at different molar ratios. The systems were prepared by gently mixing the two components at the given molar ratio. The mixture was heated to 40 °C, under constant stirring, until a clear liquid solution was formed.

### Thermal Properties - differential scanning calorimetry (DSC)

The DSC experiments were performed in a TA instrument DSC Q100 model (Thermal analysis & analysers, USA), using the different formulations in a TA aluminium pan. The temperature program for saturated fatty acids in powder and THEDES form was comprised of a heating step from -20 °C to 100 °C at 5 °C min^−1^, an isothermal step of 2 min at 100 °C and a cooling step to 20 °C at 5 °C min^−1^. For the thermograms of menthol based THEDES, the samples were equilibrated at 40 °C for 5 min followed by cooling to -40 °C, an isothermal period for 5 min and heating to 120 °C at a 5 °C /min. The temperature programme for IBU based THEDES comprise a heating step from -20 °C to 120 °C at 5 °C min^−1^, an isothermal step of 2 min at 120 °C and a cooling step to 20 °C at 5 °C min^−1^. All measurements were performed under a nitrogen atmosphere (purge gas flux of ca. 50 mL min^−1^).

### Solubility measurements

The solubility measurements were performed using the IBU in powder or in THEDES form (IBU: LIM at a molar ratio of 1:4 and 1:8). Briefly, an excess of API in powder and in THEDES form was added to a Hank’s Balanced Salt Solution (HBSS, ref 14025-092, Alfagene) solution (≈37 °C) and stirred for 24 hours. The API solubility was quantified by UV–vis spectroscopy at 265 nm in a microplate reader (BIO-TEK, SYNERGY HT).

### Cell culture

Human Caco-2 and HT29 cell lines were obtained from Deutsche Sammlung von Mikroorganismen und Zellkulturen (DSMZ, Germany) and American Type Culture Collection (ATCC, USA), respectively. These cell lines were cultured in RPMI 1640 medium supplemented with 10% of heat-inactivated fetal bovine serum (FBS) and 1% penincilin-strepmycin (PS). Cells were maintained at 37°C with 5% CO_2_ in a humidified incubator and routinely grown as a monolayer in 75 cm^2^ culture flasks. The cell culture medium and supplements were purchased from Invitrogen (Gibco, Invitrogen Corporation, UK).

### Cytotoxicity assay

Cytotoxicity assay was performed using confluent and non-differentiated Caco-2 cells, a model considered to assess the effect of chemical/food compounds and nano/microparticles on the intestinal function since it shares characteristics of the enterocytes^32^. The assay was performed as previously described by Rodrigues *et al*.^71^. Briefly, Caco-2 cells were seeded into 96-well plates at a density of 2 × 10^4^ cells/well and allowed to grow for 7 days, with medium renewal every 48 hours. At day 7, cells were incubated with the samples diluted in culture medium. Cells incubated only with culture medium were considered as control. After 24 hours, cells were washed once with PBS (Sigma-Aldrich, USA) and cell viability was assessed using CellTiter 96^®^ AQueous One Solution Cell Proliferation Assay (Promega, USA) containing MTS reagent, according to manufacturer’s instructions. The absorbance was measured at 490 nm using a Spark^®^ 10 M Multimode Microplate Reader (Tecan Trading AG, Switzerland) and cell viability was expressed in terms of percentage of living cells relatively to the control. At least three independent experiments were performed in triplicate.

### Antiproliferative assay in HT29 cell monolayer

Antiproliferative effect of THEDES and standard compounds was evaluated in HT29 cells, as described elsewhere^72^. Briefly, cells were seeded at a density of 1 × 10^4^ cells/well in 96-well culture plates. After 24 hours cells were incubated with different concentrations of the samples diluted in culture medium. Cells incubated only with culture medium were considered as control. Cell proliferation was measured after 24 hours using MTS, as mentioned above. Results were expressed in terms of percentage of living cells relatively to the control. At least three independent experiments were performed in triplicate.

### Cell transport

For transport studies, Caco-2 cells were seeded in 12 mm i.d. Transwell® inserts (polycarbonate membrane, 0.4 μm pore size, Corning Costar Corporation) in 12-well plates at a density of 2.24 × 10^5^ cell/mL. Cells were allowed to grow and differentiate to confluent monolayers for 21–24 days post seeding by changing the medium three times a week. Transepithelial electrical resistance (TEER) of grown cells in Transwell was measured using EVOM™ voltmeter (WPI, Germany). Only monolayers with a TEER value higher than 700 Ωcm^2^ were used for experiments. For the assays, cells were washed two times with HBSS and the compounds diluted in HBSS were added to the apical side. Transepithelial transport was followed at several time points (0, 15,30, 60, 120, 180, 240 minutes and 24 hours) where 200 μL was collected from the basolateral side. Samples were analysed by HPLC method as described above and concentration of the compounds determined. Results were expressed in terms of percentage of compounds at basolateral and apical sides and uptake by the cells. Three independent experiments were performed in duplicate.

### High-performance liquid chromatography (HPLC) analyses

HPLC analysis of samples resulting from transepithelial transport studies were performed using a Waters Alliance HPLC system (2695, Waters, Milford, MA, USA) coupled to a photodiode array detector (2996, Waters, Ireland). Separation was carried out in a reversed-phase column LiChrospher 100 RP-18 (5 µm) LiChroCART 250-4 (Merck Millipore, Kenilworth, NJ, USA) in isocratic mode, with a mobile phase formed by 60:40 (%v/v) acetonitrile:water acidified with phosphoric acid (pH 2.5). The injection volume was set at 40 µL, the flow rate at 1.0 mL/min and the column temperature at a constant temperature of 40°C. Transepithelial transport was followed as a function of time by detection of IBU at 221 nm. Empower Pro (2002) software was used for data acquisition.

### Cell cycle assessment

HT29 cell line was seeded at a density of 1 × 10^6^ cells in a 25 cm^2^ culture flask for 24 hours. Then, cells were incubated in different concentrations of the samples diluted in culture medium and culture medium alone (as control) for 24 hours. For flow cytometry analysis of DNA content, bare nuclei were prepared as described elsewhere^73^. Briefly, cells were detached from the culture flasks with trypsin-EDTA 0.5% and washed one time with cold PBS. Finally, 1 × 10^6^ cells were re-suspended in 1 mL of staining solution containing 50 mg/mL of Propidium Iodide (Sigma-Aldrich, USA), 1.5% (v/v) of Triton X-100 (Sigma-Aldrich, USA), 0.7 U/mL of DNase/protease-free Ribonuclease A (Thermo Scientific, USA) and 0.01 M of NaCl, followed by incubation at room temperature in the dark for 2 hours. Cell cycle was assessed by flow cytometry, using a CyFlow space (Partec GmbH) instrument, registering 30.000 events/sample as described previously^72^.

### Caspase-3 activity induction

Apoptosis was evaluated by caspase-3 activity using NucView488TM and MitoView633TM Apoptosis Assay Kit (Biotium, USA) that allows the staining of living cells with a far-red fluorescent dye MitoView633TM, while the intracellular caspase-3/7 activity and cells nuclei morphological changes were stained with bright green fluorescent dye NucView488TM. HT29 cells were seeded at a density of 40.000 cell/cm^2^ at 24-well plate for 24 hours. Then, cells were incubated another 24 hours with different concentrations of the samples diluted in culture medium and culture medium alone (as control). Staining was performed with 200 μL/well of culture medium containing 1 μL of NucView488TM and 1 μL of MitoView633TM for 2 hours. Cells were observed at fluorescence microscope (Leica DM6000, Germany) and image analysis was performed using ImageJ software.

### Measurement of cellular ROS production

Intracellular ROS level were assessed adapting the method described by Wolf and Liu^74^. HT29 cells were seeded at a density of 40.000 cell/cm^2^ in a 24-well plate. After 24 hours, cells were incubated for 1 hour with different concentrations of the samples diluted in culture medium and culture medium alone (as control). Cells were then washed two times with PBS and 600 μL of diclhorofluorescin-diacetate (DCFH-DA 25 μM) was added for 1 hour. Fluorescence was measured using a Microplate Fluorimeter FLx800 (Bioteck Instruments) (excitation and emission wavelengths of 485 nm and 528 nm, respectively). Results were expressed in terms of percentage of fluorescence intensity relatively to the control. Three independent experiments were performed in triplicate.

### Griess assay

In order to evaluate the inhibition of nitric oxide (NO) species produced by HT29 treated with THEDES and isolated compounds, culture media was assessed using Griess reaction, as previously reported^75^. A mixture of 50:50 (v/v) of Griess reagent (1% sulphanilamide, 0.1% N-(L-naphthyl)-ethylene diamine dihydrochloride and 2% H_3_PO_4_) and cell supernatant was performed, and colour developed measured at 540 nm using a Spark^®^ 10 M Multimode Microplate Reader (Tecan Trading AG, Switzerland). Percentage of NO produced was expressed relatively to the control. Three independent experiments were performed in triplicate.

### Statistical analysis

All data were expressed as mean ± standard errors (SD). GraphPad Prism 6 software was used to calculate EC_50_ values (the concentration of sample necessary to decrease 50% of cell population) and to analyse significant differences between data set through One-Way Analysis of Variance (ANOVA) following Dunnett’s multiple comparison tests. A p-value < 0.05 was considered significant.
